# Cytoplasmic Determination of Meiotic Spindle Size Revealed by a Unique Inter-Species Germinal Vesicle Transfer Model

**DOI:** 10.1038/srep19827

**Published:** 2016-01-27

**Authors:** Zhong-Wei Wang, Guang-Li Zhang, Heide Schatten, John Carroll, Qing-Yuan Sun

**Affiliations:** 1 State Key Laboratory of Reproductive Biology, Institute of Zoology, Chinese Academy of Sciences, Beijing 100101, China; 2 Department of Veterinary Pathobiology, University of Missouri, Columbia, MO 65211, USA; 3 Faculty of Medicine, Nursing and Health Sciences, Monash University, Clayton Campus, 3800, Australia

## Abstract

Spindle sizes are different in diverse species and cell types. In frogs, the meiotic spindle size is positively correlated with the egg cell volume. Across species, relatively small mouse oocytes (70–80 μm) have a relatively large spindle while larger pig oocytes (about 120 μm) have a considerably smaller spindle. In this study we investigated whether species-specific oocyte spindle size was determined by cytoplasmic or nuclear factors. By exchanging the germinal vesicle between mouse and pig oocytes, we obtained two kinds of reconstructed oocytes: one with mouse ooplasm and pig GV (mCy-pGV oocyte), and the other with pig ooplasm and mouse GV (pCy-mGV oocyte). We show that the MII spindle size of the mCy-pGV oocyte is similar to that of the mouse meiotic spindle and significantly larger than that of the pig meiotic spindle. The timing of oocyte maturation also followed that of the species from which the oocyte cytoplasm arose, although some impact of the origin of the GV was observed. These data suggest that spindle size and the timing of meiotic progression are governed by cytoplasmic components rather than cytoplasmic volume and GV materials.

Oocyte maturation is a co-ordinated process involving nuclear (cell cycle) progression and cytoplasmic changes. *In vitro* oocyte developmental time courses are different in diverse species; for example, mouse oocytes take about 10–12 h, bovine oocytes require 20 h and pig oocytes require about 44 h to reach the MII stage^1^. The sizes of mouse and pig oocytes as well as the sizes and shapes of meiotic spindles of mouse oocytes and pig oocytes are significantly different. The smaller mouse oocyte contains an elongated spindle, while the larger pig oocyte contains a barrel-shaped small spindle. The mechanisms controlling spindle size and time course of meiotic maturation are unknown but can be addressed using oocyte reconstruction techniques. In mammals, oocytes are arrested naturally in the first prophase with a very large nucleus called the germinal vesicle (GV). GV material is important for oocyte development and subsequent cell reprogramming of the early embryo^2^,^3^,^4^. GV transfer technology has been used to rescue age-related aneuploidy in oocytes^5^,^6^. Previous work by others and us has shown that the reconstructed oocytes can be matured and fertilized by intracytoplasmic sperm injection (ICSI), which leads to live animals in some species such as the mouse^7^ and rabbit^8^.

Thus, we have used the GV transfer technique, exchanged the GVs between mouse oocytes and pig oocytes to investigate the nuclear-cytoplasm interactions (Fig. 1), and to determine what controls meiotic time course and meiotic spindle size.

## Results

### *In vitro* developmental time course and spindle size of mouse and pig oocyte

**Figure 2:**
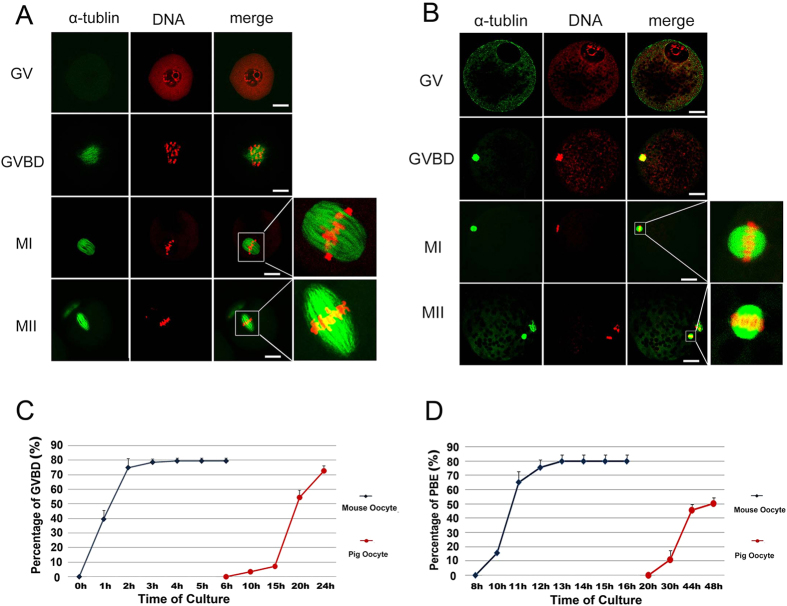
Overview of the difference in spindle size and *in vitro* maturational time course in pig and mouse oocytes. (**A)** Mouse oocytes at the GV, GVBD, MI and MII stages were stained with PI and anti-α-tublin antibody to detect mouse meiotic spindle shape and size. (**B)** Pig oocytes at the GV, GVBD, MI and MII stages were stained with PI and anti-α-tubulin antibody to detect pig meiotic spindle shape and size. (**C)** Percentages of mouse oocyte GVBDs (0 h: 0%; 1 h: 39.53 ± 6.10%; 2 h: 74.49 ± 6.26%; 3 h: 78.57 ± 2.18%; 4 h: 79.41 ± 2.03%; 5 h: 79.41 ± 2.03%; 6 h: 79.41 ± 2.03%) were observed each hour from 0–6 h of *in vitro* culture, and percentages of pig oocyte GVBDs (6 h: 0%; 10 h: 3.53 ± 1.22; 15 h: 7.11 ± 1.24; 20 h: 54.44 ± 5.09; 24 h: 72.67 ± 3.48) were observed at 6 h, 10 h, 15 h, 20 hand 24 h of *in vitro* culture. (**D)** Percentages of mouse oocytes with first polar body extrusion (8 h: 0%; 10 h: 15.70 ± 0.93%; 11 h: 65.22 ± 7.25%; 12 h: 75.56 ± 5.18%; 13 h: 79.94 ± 4.38%; 14 h: 79.94 ± 4.38%; 15 h: 79.94 ± 4.38%; 16 h: 79.94 ± 4.38%) were observed one or two hours from 8 h to 16 h of *in vitro* culture, and percentages of pig oocytes with first polar body extrusion (20 h: 0%; 30 h: 11.01 ± 6.27%; 44 h: 45.55 ± 3.85%; 48 h: 50.14 ± 4.07%) were observed at 20 h, 30 h, 44 hand 48 h of *in vitro* culture (bar = 30 μm).

Mouse and pig oocytes were stained by PI and anti-α-tubulin antibody to detect chromosome condensation and spindle assembly. MI and MII spindles of mouse oocytes were larger than the spindles of pig oocytes (Fig. 2A,B). Mouse oocyte GVBD took place in less than 4 h of *in vitro* culture and first polar body extrusion took place at 10–13 h of *in vitro* culture. However, the pig oocytes took 10–24 h to undergo GVBD and 30–48 h for first polar body extrusion (Fig. 2C,D).

### Maturation of the mCy-pGV oocyte reconstructed by fusing mouse cytoplast with pig GV

**Figure 3:**
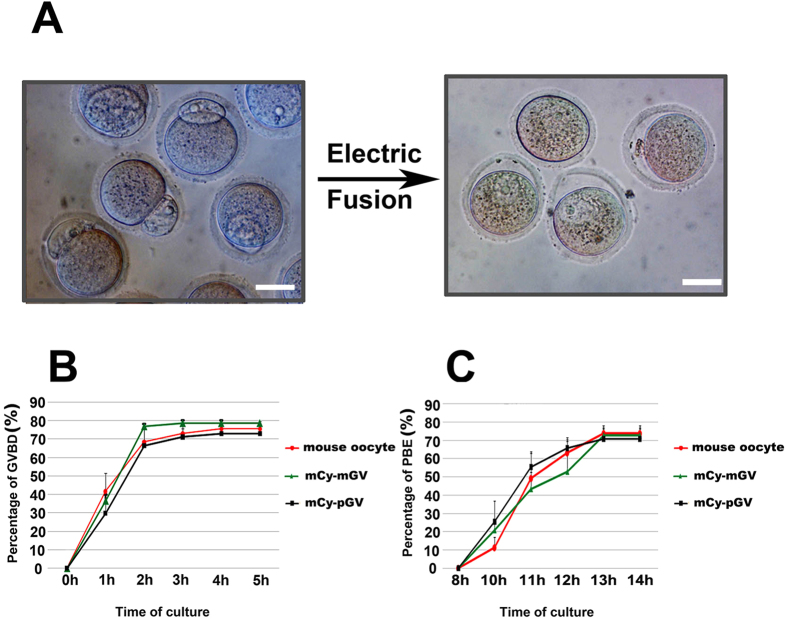
*In vitro* maturational time course is similar between mCy-pGV oocytes and mouse oocytes. (**A**) Pig GV was fused to the mouse enucleated oocyte by electric fusion. (**B**) Percentages of GVBD in mouse oocytes (0 h: 0%; 1 h: 41.61 ± 11.35% 2 h: 68.51 ± 9.84%; 3 h: 73.11 ± 6.68%; 4 h: 75.53 ± 2.57%;5 h: 75.53 ± 2.57%), mCy-mGV oocytes (0 h: 0%; 1 h: 36.62 ± 0.24%; 2 h: 76.99 ± 3.17%; 3 h: 78.74 ± 1.38%; 4 h: 78.74 ± 1.38%; 5 h: 78.74 ± 1.38%) and mCy-pGV oocytes (0 h: 0%; 1 h: 29.81 ± 6.05%; 2 h: 66.37 ± 12.46%; 3 h: 71.64 ± 8.91%; 4 h: 72.92 ± 6.82%; 5 h72.91 ± 6.82%) were observed each hour from 0 to 5 h of *in vitro* culture. (**C**) Percentages of mouse oocytes (8 h: 0%; 10 h: 10.57 ± 4.41%; 11 h: 49.17 ± 13.72%; 12 h: 63.10 ± 8.22%; 13 h: 73.69 ± 3.04%; 14 h: 73.69 ± 3.04%), mCy-mGV oocyte(8 h: 0%; 10 h: 20.88 ± 3.76%; 11 h: 43.46 ± 8.90%; 12 h: 52.65 ± 5.84%; 13 h: 72.71 ± 5.28%; 14 h: 72.71 ± 5.29%) and mCy-pGV oocytes (8 h: 0%; 10 h: 25.56 ± 11.16%; 11 h: 55.27 ± 8.33%; 12 h: 65.65 ± 3.71%; 13 h: 70.78 ± 5.42%; 14 h: 70.78 ± 5.42%) with first polar body extrusion were detected 8 h, 10 h, 12 h, 13 h, and 14 h of *in vitro* culture (bar = 30 μm).

Mouse and pig oocytes were enucleated by micro-manipulation, and then the pig GV was transferred into the perivitelline space of the enucleated mouse oocyte. The pig GV and mouse cytoplast were fused by electric fusion (Fig. 3A). Mouse GV and mouse cytoplast were also fused as a mock control (mCy-mGV oocytes). mCy-pGV oocytes were cultured *in vitro* and observed every 1 or 2 hours from 0 h to 14 h. The developmental time course of the mCy-pGV oocytes was similar to that of the mouse oocytes and mCy-mGV oocytes (Fig. 3B,C). The mCy-pGV oocytes underwent GVBD within 4 h, and the first polar body extrusion took place at 10–13 h after release from milrinone inhibition. Percentages of the oocyte GVBD and the first polar body extrusion were not significantly different between the mCy-pGV oocytes, control mouse oocytes and mCy-mGV oocytes (Fig. 3B,C).

### Spindle size of the mCy-pGV oocyte reconstructed by fusion of mouse cytoplast with pig GV

**Figure 4:**
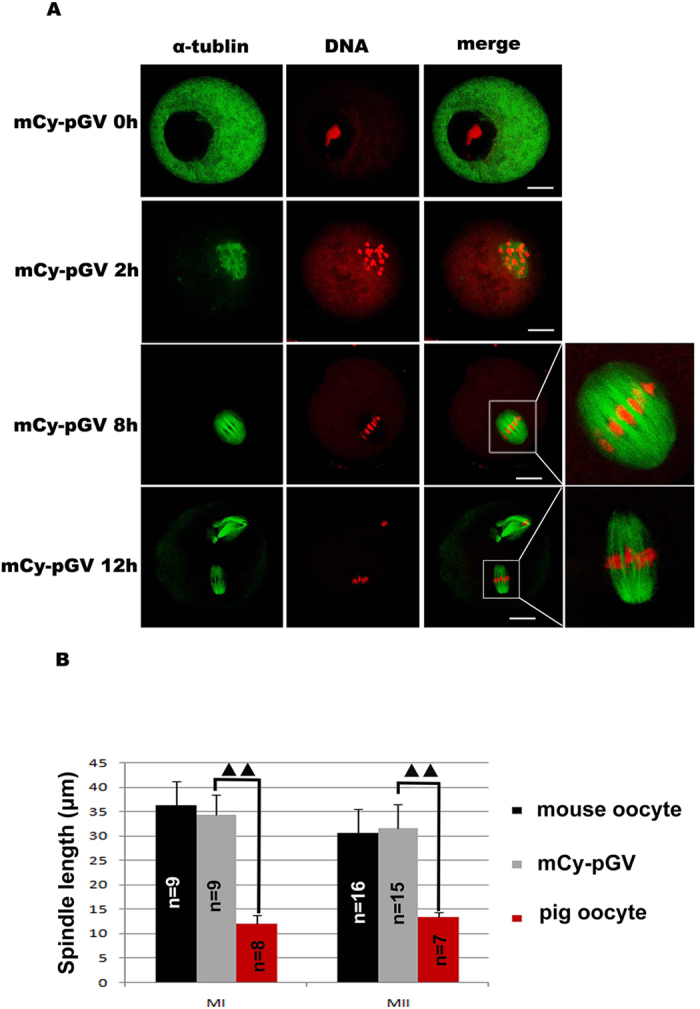
Spindle size of mCy-pGV oocytes. (**A)** mCy-pGV oocytes were stained with PI and anti-α-tubulin antibody at 0 h(GV stage), 2 h(GVBD stage), 8 h(MI stage) and12 h(MII stage) of culture. (**B)** Meiotic spindle size was measured; the sizes of MI (34.25 ± 4.17 μm) and MII (31.57 μm ± 4.90 μm) spindles of the mCy-pGV oocytes were similar to those of MI (36.23 ± 4.99 μm) and MII (30.50 ± 4.91 μm) spindles of mouse oocytes, but significantly larger than those of the pig oocytes (MI: 11.94 ± 1.80 μm; MII: 13.32 ± 1.10 μm) (bar = 30 μm).

In mCy-pGV oocytes, normal chromosome condensation could be detected and spindles were well assembled in both MI and MII stages. The size of spindles of mCy-pGV oocytes was similar to that of the mouse oocytes but significantly larger than that of the pig oocytes (Fig. 4A). The sizes of the MI and MII spindles in mCy-pGV oocytes were 34.25 ± 4.17 μm and 31.57 μm ± 4.90 μm, respectively, which were similar to those of the mouse MI oocytes (36.23 ± 4.99 μm) and MII oocytes (30.50 ± 4.91 μm), but significantly larger than those of pig MI oocytes (11.94 ± 1.80 μm) and MII oocytes (13.32 ± 1.10 μm) (*p* < *0.05*) (Fig. 4B).

### Maturation of the pCy-mGV oocyte reconstructed by microinjection of mouse GV into pig cytoplast

**Figure 5:**
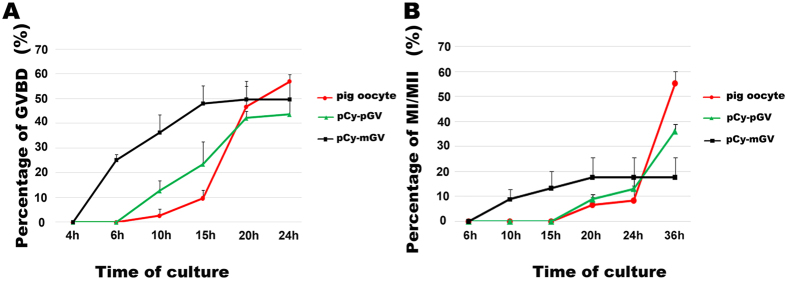
*In vitro* maturational time course of pig oocyte and pCy-mGV oocytes. (**A**) Percentages of GVBD in pig oocytes (4 h: 0%; 6 h: 0%; 10 h: 3.54 ± 1.62%; 15 h: 9.71 ± 3.31%; 20 h: 46.73 ± 8.44%; 24: 56.94 ± 2.98%), pCy-pGV oocytes (0 h: 0%; 6 h: 0%; 10 h: 12.58 ± 3.09%; 15 h: 23.48 ± 9.19%; 20 h: 42.12 ± 2.92%; 24 h: 43.64 ± 5.53%) and pCy-mGV oocytes (4 h: 0%; 6 h: 25.24 ± 2.20%; 10 h: 36.27 ± 7.17%; 15 h: 48.19 ± 7.11%; 20 h: 49.77 ± 7.44%; 24: 49.77 ± 7.44%) were observed at 4 h, 6 h, 10 h, 15 h, 20 hand 24 h of *in vitro* culture. pCy-mGV oocytes reached the GVBD stage at 6–20 h of culture which was 4 hearlier than the pig oocytes (20–24 h). B: Percentages of pig oocytes (6 h: 0%; 10 h: 0%; 15 h: 0%; 20 h: 6.67 ± 2.89%; 24 h: 8.33 ± 5.77%; 36 h: 55.00 ± 5.00%), pCy-pGV oocytes (6 h: 0%; 10 h: 0%; 15 h: 0%; 20 h: 8.86 ± 2.04%; 24 h: 13.10 ± 4.29%; 36 h: 35.98 ± 2.79%) and pCy-mGV oocytes (6 h: 0%; 10 h: 8.75 ± 3.98%; 15 h: 13.19 ± 6.88%; 20 h: 17.50 ± 7.95%; 24 h: 17.50 ± 7.95%; 36 h: 17.50 ± 7.95%) reaching the MI stage were detected at 6 h, 10 h, 15 h, 20 h, 24 hand 36 h of *in vitro* culture. pCy-mGV oocytes reached the MI stage at 10–20 h of *in vitro* culture which was 10 hearlier than the pig oocyte (20–36 h).

Mouse GV was directly injected to the pig enucleated oocyte using a piezo unit. GVBD of the pCy-mGV oocytes took place at 6–20 h of culture *in vitro*, which was significantly extended compared to the time course of mouse oocyte GVBD, although it was 4 hours earlier than that of the pig oocyte GVBD (10–24 h) (Fig. 5A). The reconstructed oocytes developed to the MI stage at 10–20 h of *in vitro* culture, which was significantly longer than in mouse oocytes, but 10 hearlier than the pig oocytes reaching the MI stage (20–36 h) (Fig. 5B). However, probably due to the removal of cumulus cells, the reconstructed oocytes failed to develop to the MII stage in our culture system. It has been previously reported that the pig oocytes without surrounding cumulus cells have a limited developmental capability^9^. Pig GV and pig cytoplast were fused as a mock control (pCy-pGV oocytes), which also had a compromised maturational capability, and failed to reach the MII stage (Fig. 5B).

### Spindle size of pCy-mGV oocyte reconstructed by microinjection of mouse GV into pig GV cytoplast

**Figure 6:**
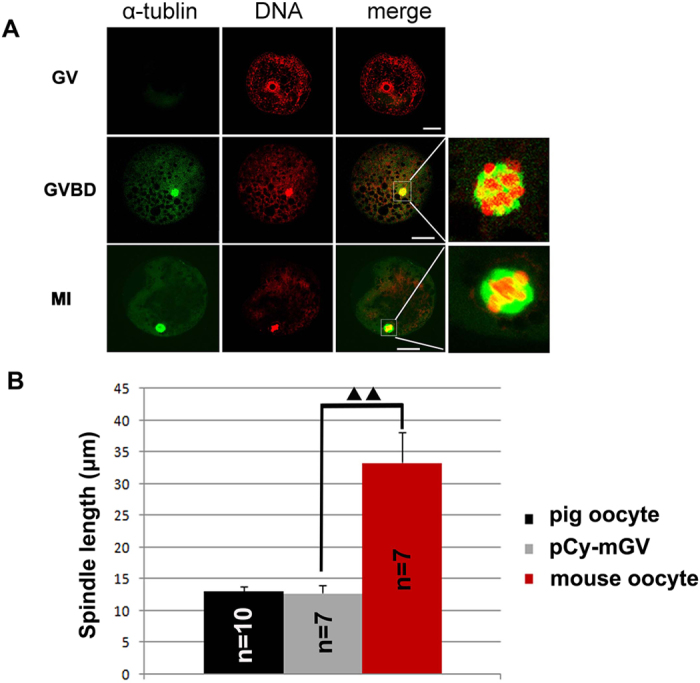
Spindle size of pCy-mGV oocytes. (**A)** pCy-mGV oocytes at GV, GVBD and MI stages were stained with PI and anti-α-tubulin antibody. Normal chromosome condensation could be detected in GVBD stage oocytes. A small spindle was assembled in the pCy-mGV oocytes. (**B)** Spindle size of the MI stage pCy-mGV oocytes were12.80 ± 1.27 μm, significantly small than that of mouse oocytes (33.33 ± 4.80 μm) (*p* < *0.05*), but similar to that of pig oocytes (13.17 ± 0.74 μm) (bar = 30 μm).

Chromosomes condensed normally when pCy-mGV oocytes reached the GVBD stage at 6–10 h of *in vitro* culture (Fig. 6A). A small spindle was assembled in the reconstructed pCy-mGV oocyte at the MI stage (Fig. 6A). Spindle size of the pCy-mGV oocyte was 12.80 ± 1.27 μm, which was similar to that of the pig MI spindle (13.17 ± 0.74 μm) and significantly shorter than that of the mouse MI spindle (33.33 ± 4.80 μm) (Fig. 6B).

## Discussion

The spindle is an important cellular structure that functions to segregate chromosomes in both mitotic and meiotic cells. Spindle size is determined by a series of microtubule-associated proteins as well as the cell size. Molecular mechanisms of spindle length control in mitotic cells have been widely studied. The balance between microtubule (MT) polymeriztion and depolymerization is critical for the spindle length control^10^,^11^. Spindle size decreases or stops growing after inhibition of MT polymerization factors such as TOG^12^, EB1^13^,CLASP^14^. However, knockdown of the MT depolymerization-related proteins such as, kinesin-8^15^ and kinesin-13^16^ results in elongated and monopolar spindles. The microtubule-severing ATPase katanin is also important in mitotic and meiotic spindle length control. Inhibition of katanin severing activity reduces nocodazole-induced spindle microtubule depolymerization^17^,^18^. The spindle size also varies greatly to accommodate rapid changes in cell size. In *Xenopus*, the diameter of the *X. laevis* eggs is 1.2 mm which is five-fold large than the *X. tropicalis* eggs (diam ~ 0.6 mm), and the spindle of the *X. laevis* is larger, too^19^. Two recent studies showed that cell volume is related to spindle size in mitotic *Xenopus* embryo cells, and changes in cytoplasmic volume are sufficient to drive spindle scaling.^20^,^21^. Thus, availability of cytoplasmic components, together with cell size, determines spindle size in mitotic embryonic cells.

On the other hand, nuclear materials are quite important for cellular activities. If the GV was replaced by primary spermatogonium, fibroblast or cumulus cells the reconstructed oocyte could not adequately complete the meiotic cell cycle^22^,^23^. The cytoplasm of GV stage oocytes^3^ and pronuclear stage zygotes^24^,^25^ were not suitable for nuclear transfer experiments. Enucleated MII oocytes, the cytoplasm of metaphase zygotes and metaphase blastomeres of 2-cell embryos can successfully reprogram the somatic cell, suggesting that the material enclosed in the pronucleus may be involved in the successful reprogramming, However, a recent investigation using the interphase cytoplasm of two-cell mouse embryos as the nuclear transfer recipients resulted in live offspring^26^. This suggests that the materials in the germinal vesicle and the pronuclei may be more important than in the somatic cell (blastomere of 2-cell embryo) nucleus. In addition, the nucleolus of the GV stage oocyte which is localized in the germinal vesicle is essential for nucleolus formation of the early embryo^4^, which explains the importance of the GV materials for the oocyte and subsequent embryo.

Interestingly, the pig oocyte has a relatively large cell size compared to the mouse oocyte. However, the spindle size of the pig oocyte is much smaller than that of the mouse oocyte. In addition, fully grown pig oocytes need much longer time to resume meiosis and complete meiotic maturation compared to mouse oocytes. We wondered if the germinal vesicle material or the cytoplasm plays critical functions in determining the spindle size and meiotic maturation time course in meiotic mammalian oocytes.

**Figure 7:**
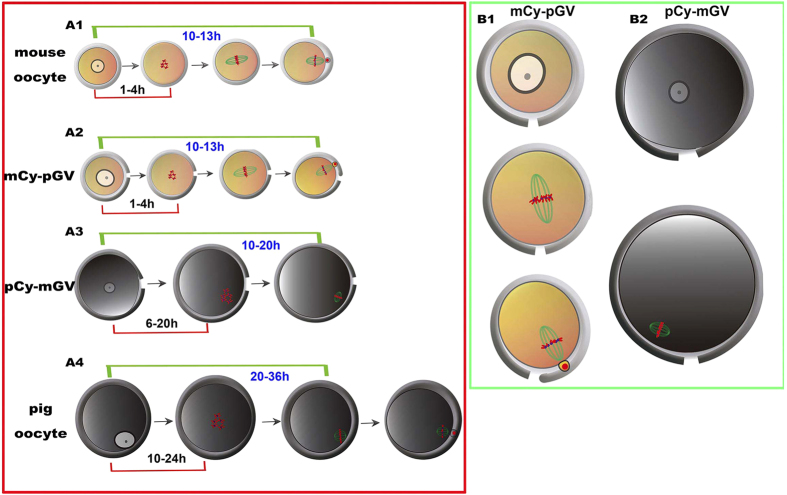
Summary of *in vitro* maturational time course and spindle size determination in mCy-pGV and pCy-mGV oocytes. (**A1)** Normal mouse oocytes take 1–4 h for GVBD and 10–13 h for PBE. (**A2)** mCy-pGV oocytes take 1–4 h for GVBD and 10–13 h for PBE, which is similar to the mouse oocyte *in vitro* maturational time course. (**A3)** pCy-mGV oocytes take 6–20 h to reach GVBD, and 10–20 h to reach the MI stage, which is significantly longer than that of mouse oocytes; (**A4)** Pig oocytes take 10–24 h for GVBD and 20–36 h for PBE, which is longer than that of *in vitro* maturation of pCy-mGV oocytes. (**B1)** mCy-pGV oocytes contain a large spindle similar to mouse oocyte spindles. (**B2)** pCy-mGV oocytes contain small spindles similar to the pig oocyte spindles.

Sendai-virus-mediated fusion of GV-stage oocyte was used to investigate the interaction between the germinal vesicle and cytoplasm^27^,^28^,^29^. After cell fusion, it was found that the MPF (maturation-promoting factor) function was not species-specific. However, this cell fusion approach could not distinguish between the cytoplasmic and nuclear influence. We thus used GV transfer technology to reconstruct the oocyte with GV and cytoplast derived from mouse and pig oocytes. In the mCy-pGV and pCy-mGV oocytes, the spindle can be assembled normally. mCy-pGV oocytes can develop to the MII stage like mouse oocytes. The pCy-mGV oocyte has a compromised maturational capability compared to the pig oocyte, which may be a result of the removal of the granulosa cells surrounding the oocyte before micro-manipulation. Centrifugation of the pig oocytes before enucleation of the oocyte may be another reason for the weak maturational capability. Although the pCy-mGV oocyte cannot develop to the MII stage, the MI spindle assembled normally in the pCy-mGV oocyte. Interestingly, when the mouse oocyte GV was transferred into the enucleated pig oocyte, a small barrel-shaped spindle was formed, while when the pig oocyte GV was transferred into the enucleated mouse oocyte, a large spindle was formed (Fig. 7). Our results suggest that it is the cytoplasmic components rather than the GV materials that determine the meiotic spindle size and shape in mammalian oocytes.

The maturational time course of oocytes was also largely determined by the ooplasm. The mCy-pGV oocyte has a considerable developmental capability like the mouse oocyte. The percentage of GVBD and the maturational time course were similar to the mouse oocyte. Most mCy-pGV oocytes underwent GVBD at the same time as mouse oocytes and could develop to the MII stage at 10–13 h of *in vitro* culture. When the GV of a mouse oocyte was transferred into an enucleated pig oocyte, the *in vitro* developmental time to GVBD and the MI stage were also significantly extended. GVBD of the pCy-mGV oocyte took place at 6–20 h of *in vitro* culture, which is much slower compared to mouse oocyte GVBD, but 4 h faster than pig oocyte GVBD (10–24 h) (Fig. 7). Therefore, the oocyte meiotic maturational time course is mainly determined by the cytoplasm, while the GV materials may also regulate this process to a certain extent.

## Conclusions

The spindle sizes of mCy-pGV oocytes and pCy-mGV oocytes are both similar to those of oocytes which contribute the cytoplasm. Unlike in *Xenopus* mitotic embryonic cells, it is the ooplasmic components but not the cytoplasmic volume that determine the spindle size in mammalian oocytes. Although GV materials are important for somatic cell reprogramming and subsequent development of the embryo, it is not related to spindle size control.

## Methods

### Reagents and animals

All reagents were purchased from Sigma unless otherwise noted. All mice were housed under specific pathogen-free controlled conditions with a 14L:10D light/dark cycle. The animal maintenance, handling and all the relevant details in the methods section were carried out in accordance with the Institutional Guidelines for Animal Use and Care at the Institute of Zoology, Chinese Academy of Sciences. All experimental protocols were approved by the ethical committee of the Institute of Zoology, Chinese Academy of Sciences (approval number IOZ12017).

### Mouse oocyte collection and *in vitro* maturation

Immature oocytes at the germinal vesicle (GV) stage were collected from ovaries of 6–8-wk-old ICR female mice in M2 medium. Oocytes used for micro-manipulation were cultured in M2 medium supplemented with 2.5 μM milrinone to maintain the oocytes at the GV stage during micro-manipulation. After micro-manipulation, oocytes were washed thoroughly and further cultured in M16 medium covered with liquid paraffin oil at 37 °C in an atmosphere of 5% CO_2_ in air until proceeding to the GV, GVBD, MI and MII stages.

### Pig oocyte collection and *in vitro* maturation

Collection and *in vitro* maturation of pig oocytes were conducted as previously reported^30^. In brief, pig ovaries were obtained from our local abattoir and transported to the laboratory at 35 °C. Cumulus-oocytes complexes (COCs) were aspirated from antral follicles (3–6 mm in diameter) of ovaries with an 18-gauge needle fixed to a 20 ml disposable syringe. After three rinses in washing medium (TCM-199 medium (Invitrogen) supplemented with 2.2% NaHCO_3_), COCs with uniform cytoplasm and at least four layers of compact cumulus cells were selected for culture. To prepare cumulus cell-denuded oocytes (DOs), cumulus cells were freed from the collected COCs after treatment for 3 min with 300 IU/ml hyaluronidase, followed by repeated pipetting using a narrow-bore micropipette. COCs and DOs were cultured in the basic medium with hormone. Basic medium was TCM-199, supplemented with 0.1% PVA (w/v), 0.91 mM sodium pyruvate, 75 μg/ml potassium penicillin G and 50 μg/ml streptomycin sulphate. Pig oocytes were cultured in basic medium with 0.57 mM cysteine, 0.5 μg/ml FSH, 0.5 μg/ml LH, and 10 ng/ml EGF. 4 mM HX was added to the culture medium to prevent the oocytes from GVBD for micro-manipulation. Oocytes of the control group or after micro-manipulation were cultured in 500 μl maturation medium in 4-well dishes, covered by liquid paraffin oil for up to 44 h at 38.5 °C in an atmosphere of 5% CO_2_ in air and saturated humidity.

### Preparation of mouse GVs and cytoplasts

Germinal vesicles and cytoplasts for GV transfer were obtained as described in previous reports^6^,^31^. GV stage oocytes were kept in M2 medium supplemented with 2.5 μM milrinone to maintain the oocytes at the GV stage. Then the oocytes were cultured in M16 medium supplemented with 2.5 M milrinone for 2 h. After 2 h of culture in milrinone-supplemented M16 medium, GV oocytes have developed a perivitelline space, facilitating manipulation and enucleation. Enucleation and GV transplantation were performed in manipulation drops of M2 medium supplemented with 2.5 μM milrinone, 5 μg/ml cytochalasin B (CB) and 0.2 mg/ml demecolcine (M-Enu-M2). Immature oocytes were placed in manipulation drops for 30 min before GV removal. A slit was made in the zona pellucida by pressing a sharp glass needle through the perivitelline space against the holding pipette (See Supplementary file 1). A blunt-tip micropipette with an inner diameter of about 20 μm was inserted to remove the GV (See Supplementary file 2). The micropipette was rinsed in 10% PVP before GV removal to reduce sticking of the membranes within the pipette, then two or three droplets of mineral oil were aspirated to help control the fluid flow during the manipulation. Mouse GVs and enucleated mouse oocytes (cytoplasts) were placed in the milrinone-supplemented M16 medium for further manipulation.

### Preparation of pig GVs and cytoplasts

The protocol of pig GV removal was based on previous reports of pig^32^ and bovine^33^ oocyte GV transfer procedure with some modifications. Pig DOs were exposed to basic medium supplemented with 15 μg/ml CB and 0.1 μg/ml demecolcine for 30 min at 38.5 °C, with 5% CO_2_ in air. Micro-manipulation of pig oocyte GV transfer was carried out in P-Enu-M2 medium which was M2 medium supplemented with 15 μg/ml cytochalasin B and 0.1 μg/ml demecolcine. Pig oocytes were washed several times, transferred into P-Enu-M2, and centrifuged for 10 min at 4000 g for visualization of the GV. Then a slit was made in the zona pellucida by pressing a sharp glass needle through the perivitelline space against the holding pipette near the position of the germinal vesicle. The germinal vesicle was then squeezed out by pressing on the zona pellucida (See Supplementary file 3). Oocytes were then carefully pipetted to separate the removed GV and the oocyte.

### GV transfer by Piezo manipulation and electric fusion

A pig or mouse GV was transferred into the perivitelline space of a pig or mouse cytoplast by a blunt-tip micropipette with an inner diameter of about 25 μm (for mouse GV transfer) or 30 μm (for pig GV transfer). The pig GV-mouse cytoplast complexes (mouse GV-mouse cytoplast, pig GV-pig cytoplast) were transferred to a drop of fusion medium (0.3 M mannitol, 0.1 mM CaCl_2_ and 0.05 mM MgSO_4_ in sterile water). Electro-fusion was stimulated with two electrical pulses (160 V/mm DC for 60 ms) delivered by a Kefa Electro Cell manipulator (Academia Sinica). The fusion rate was examined 30 min later. The mouse GV was directly injected into pig enucleated oocyte cytoplasm (cytoplast) by a piezo-actuated micromanipulator. First, a single weak piezo pulse was applied to break the cytoplasmic membrane around the GV in a 20 μm pipette. The pipette was inserted from the incision of the zona pellucida, and advanced until it almost reached the opposite side of the oocyte. One weak piezo pulse was applied to puncture the oolemma at the pipette tip, and the mouse GV was immediately expelled into the enucleated pig oocyte (cytoplast) (See Supplementary file 4).

### Antibodies and immunofluorescence

Oocytes were first fixed in 4% paraformaldehyde for 30 min. The permeabilization procedures were different in diverse oocytes: mouse or mCy-pGV oocytes were treated with 0.5% Triton X-100 for 20 min at room temperature; pig or pCy-mGV oocytes were treated with 0.5% Triton X-100 over night at 37 °C covered with mineral oil. After blocking in 1% BSA for 1 h, the oocytes were further washed four times in PBS with 0.05% Tween 20 and stained with anti-α-tubulin antibody (1:100 in PBS with 0.05% Tween 20, Sigma) over night at 4 °C. After four washes in PBS with 0.05% Tween 20, the oocytes were incubated with PI (propidium iodide) in PBS containing 0.05% Tween 20 for 10 min. Finally, the oocytes were mounted on glass slides and examined with a laser scanning confocal microscope (Zeiss LSM 510 and 710 META). Spindle sizes were measured by the software ZEN 2009 Light Edition (Carl Zeiss MicroImaging GmbH).

### Statistical analysis

All experiments used at least three independent samples and were repeated at least three times; results are given as means  ±  SD. Group comparisons were made by two-tailed unpaired Student’s t-tests. P-values of <0.05 were considered significant.

## Additional Information

**How to cite this article**: Wang, Z.-W. *et al.* Cytoplasmic Determination of Meiotic Spindle Size Revealed by a Unique Inter-Species Germinal Vesicle Transfer Model. *Sci. Rep.* **6**, 19827; doi: 10.1038/srep19827 (2016).

## Supplementary Material

Supplementary file 1

Supplementary file 2

Supplementary file 3

Supplementary file 4

Supplementary Information

## Figures and Tables

**Figure 1:**
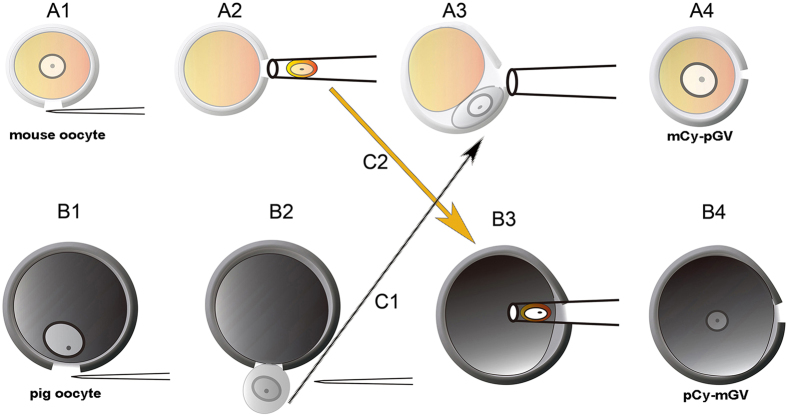
Procedure of GV transfer between mouse and pig oocytes. (**A1)** Zona pellucida of mouse oocytes was cut with a sharp glass needle. (**A2)** Mouse GV was aspirated by a blunt-tip micropipette with an inner diameter of about 20 μm. (**A3)** Pig GV was transferred to the perivitelline space of enucleated mouse oocyte by a blunt-tip micropipette with an inner diameter of about 25 μm. (**A4)** Pig GV was fused to the mouse cytoplast by electric fusion. (**B1)** Zona pellucida of mouse oocyte was cut by using pressure with a sharp glass needle. (**B2)** Pig GV was squeezed out by pressing the zona pellucida. (**B3)** Mouse GV was directly injected into pig cytoplast by a blunt-tip micropipette with an inner diameter of about 20 μm with a piezo unit. (**B4)** A pCy-mGV oocyte was reconstructed with pig cytoplast and mouse GV. (**C1)** Pig GV was transferred to the micro-manipulation droplet with enucleated mouse oocyte. (**C2)** Mouse GV was transferred to the micro-manipulation droplet with enucleated pig oocyte.
