# Supplementary material for: Cytoplasmic Determination of Meiotic Spindle Size Revealed by a Unique Inter-Species Germinal Vesicle Transfer Model
Source: Sci Rep. 2016 Jan 27;6:19827. doi: 10.1038/srep19827 (PMC4728387; doi:10.1038/srep19827)
Supplement: Supplementary Information [file srep19827-s5.pdf]

Cytoplasmic Determination of Meiotic Spindle Size Revealed by a Unique Inter-Species Germinal Vesicle Transfer Model

Zhong-Wei Wang[1], Guang-Li Zhang[1], Heide Schatten[2], John Carroll[3], Qing-Yuan Sun[1]*

[1]State Key Laboratory of Reproductive Biology, Institute of Zoology, Chinese Academy of Sciences, Beijing 100101, China

[2]Department of Veterinary Pathobiology, University of Missouri, Columbia, MO 65211, USA

[3]Faculty of Medicine, Nursing and Health Sciences, Monash University, Clayton Campus, 3800, Australia

*Corresponding author

Supplementary file 1. **A slit was made in the mouse oocyte zona pellucida**

Supplementary file 2. Mouse GV was removed by a blunt-tip micropipette

Supplementary file 3. Pig GV was squeezed out from the slit of the zona pellucida

Supplementary file 4. Mouse GV was injected to the pig cytoplast by a piezo-actuated micromanipulator
